# Dectin-1-Targeted Antifungal Liposomes Exhibit Enhanced Efficacy

**DOI:** 10.1128/mSphere.00025-19

**Published:** 2019-02-13

**Authors:** Suresh Ambati, Aileen R. Ferarro, S. Earl Khang, Jianfeng Lin, Xiaorong Lin, Michelle Momany, Zachary A. Lewis, Richard B. Meagher

**Affiliations:** a Department of Genetics, University of Georgia, Athens, Georgia, USA; b Department of Microbiology, University of Georgia, Athens, Georgia, USA; c Fungal Biology Group and Department of Plant Biology, University of Georgia, Athens, Georgia, USA; Carnegie Mellon University

**Keywords:** *Aspergillus fumigatus*, amphotericin B, antifungal agents, aspergillosis, beta-glucans, cell wall, dectin-1, experimental therapeutics, fungicidal, innate immune receptor, liposomes

## Abstract

*Aspergillus* species cause pulmonary invasive aspergillosis resulting in nearly 100,000 deaths each year. Patients at the greatest risk of developing life-threatening aspergillosis have weakened immune systems and/or various lung disorders. Patients are treated with antifungals such as amphotericin B (AmB), caspofungin acetate, or triazoles (itraconazole, voriconazole, etc.), but these antifungal agents have serious limitations due to lack of sufficient fungicidal effect and human toxicity. Liposomes with AmB intercalated into the lipid membrane (AmB-LLs; available commercially as AmBisome) have severalfold-reduced toxicity compared to that of detergent-solubilized drug. However, even with the current antifungal therapies, 1-year survival among patients is only 25 to 60%. Hence, there is a critical need for improved antifungal therapeutics. Dectin-1 is a mammalian innate immune receptor in the membrane of some leukocytes that binds as a dimer to beta-glucans found in fungal cell walls, signaling fungal infection. Using a novel protocol, we coated AmB-LLs with Dectin-1’s beta-glucan binding domain to make DEC-AmB-LLs. DEC-AmB-LLs bound rapidly, efficiently, and with great strength to Aspergillus fumigatus and to Candida albicans and Cryptococcus neoformans, highly divergent fungal pathogens of global importance. In contrast, untargeted AmB-LLs and bovine serum albumin (BSA)-coated BSA-AmB-LLs showed 200-fold-lower affinity for fungal cells. DEC-AmB-LLs reduced the growth and viability of A. fumigatus an order of magnitude more efficiently than untargeted control liposomes delivering the same concentrations of AmB, in essence decreasing the effective dose of AmB. Future efforts will focus on examining pan-antifungal targeted liposomal drugs in animal models of disease.

**IMPORTANCE** The fungus Aspergillus fumigatus causes pulmonary invasive aspergillosis resulting in nearly 100,000 deaths each year. Patients are often treated with antifungal drugs such as amphotericin B (AmB) loaded into liposomes (AmB-LLs), but all antifungal drugs, including AmB-LLs, have serious limitations due to human toxicity and insufficient fungal cell killing. Even with the best current therapies, 1-year survival among patients with invasive aspergillosis is only 25 to 60%. Hence, there is a critical need for improved antifungal therapeutics. Dectin-1 is a mammalian protein that binds to beta-glucan polysaccharides found in nearly all fungal cell walls. We coated AmB-LLs with Dectin-1 to make DEC-AmB-LLs. DEC-AmB-LLs bound strongly to fungal cells, while AmB-LLs had little affinity. DEC-AmB-LLs killed or inhibited A. fumigatus 10 times more efficiently than untargeted liposomes, decreasing the effective dose of AmB. Dectin-1-coated drug-loaded liposomes targeting fungal pathogens have the potential to greatly enhance antifungal therapeutics.

## Abstract (precis)

The fungus Aspergillus fumigatus causes pulmonary invasive aspergillosis resulting in nearly 100,000 deaths each year. Patients are often treated with antifungal drugs such as amphotericin B (AmB) loaded into liposomes (AmB-LLs), but all antifungal drugs, including AmB-LLs, have serious limitations due to human toxicity and insufficient fungal cell killing. Even with the best current therapies, 1-year survival among patients with invasive aspergillosis is only 25 to 60%. Hence, there is a critical need for improved antifungal therapeutics. Dectin-1 is a mammalian protein that binds to beta-glucan polysaccharides found in nearly all fungal cell walls. We coated AmB-LLs with Dectin-1 to make DEC-AmB-LLs. DEC-AmB-LLs bound strongly to fungal cells, while AmB-LLs had little affinity. DEC-AmB-LLs killed or inhibited A. fumigatus 10 times more efficiently than untargeted liposomes, decreasing the effective dose of AmB. Dectin-1-coated drug-loaded liposomes targeting fungal pathogens have the potential to greatly enhance antifungal therapeutics.

## INTRODUCTION

Hundreds of fungal species indigenous to our environment cause a wide variety of diseases, including aspergillosis, blastomycosis, candidiasis, cryptococcosis, coccidioidomycosis (valley fever), and *Pneumocystis* pneumonia (PCP). Collectively, pathogenic fungi infect many different organs, but lungs are the most common site for deep mycoses. Globally, aspergillosis, candidiasis, and cryptococcosis kill about 1 million or more people each year (1, 2).

Aspergillus fumigatus and related *Aspergillus* species cause aspergillosis (2). Patients at the greatest risk of developing life-threatening aspergillosis have weakened immune systems, for example, from stem cell or organ transplants, or have various lung diseases, including tuberculosis, chronic obstructive pulmonary disease, cystic fibrosis, or asthma. Among immunocompromised patients, aspergillosis is the second most common fungal infection, after candidiasis (3, 4). Additional costs associated with treating invasive aspergillosis are estimated at $40,000 per child and $10,000 per adult. Patients with aspergillosis are treated with antifungals such as amphotericin B (AmB), caspofungin, or triazoles. Even with antifungal therapy, however, 1-year survival among immunocompromised patients with aspergillosis is only 25% to 60%. Furthermore, all known antifungal agents that treat aspergillosis are quite toxic to human cells (5, 6).

AmB is the most commonly used agent for many kinds of fungal infections, including aspergillosis. Because AmB binds the fungal plasma membrane sterol ergosterol more efficiently than the mammalian sterol cholesterol, AmB is more toxic to fungal cells. The side effects of AmB include neurotoxicity and/or nephrotoxicity and/or hepatoxicity (5, 6) and can result in death of the patient (1).

**FIG 1:**
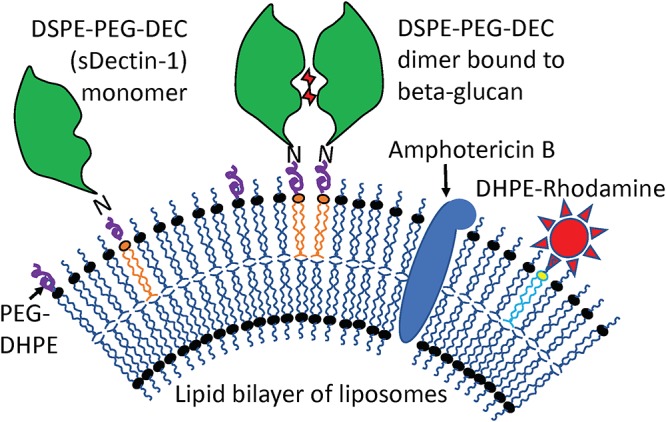
Model of DEC-AmB-LLs, liposomes loaded with sDectin-1, AmB, and rhodamine. AmB (blue oval structure) was intercalated into the lipid bilayer of 100-nm-diameter liposomes. sDectin-1 (DEC, green globular structure) was coupled to the lipid carrier DSPE-PEG. Both DSPE-PEG-DEC and red fluorescent DHPE-rhodamine (red star) were also inserted into the liposomal membrane. sDectin-1, rhodamine, AmB, and liposomal lipids were in a 1:2:11:100 mole ratio (Table S1). Two sDectin-1 monomers (two DSEP-PEG-DEC molecules) must float together in the membrane to bind strongly to cell wall beta-glucans (red sugar moieties). The two liposomal controls examined were BSA-AmB-LLs that contain equal microgram amounts of 65-kDa BSA in place of 22-kDa sDectin-1 (i.e., 0.33:2:11:100 mole ratio) and AmB-LLs lacking any protein coating (0:2:11:100 mole ratio). From these mole ratios, the surface area of an 100-nm-diameter liposome, and the published estimate of 5 × 10^6^ lipid molecules per 10^6^ nm^2^ of lipid bilayer (70), we estimated that there were approximately 3,000 rhodamine molecules in each liposome preparation and 1,500 sDectin-1 monomers in each DEC-AmB-LL. Note that for simplicity the proper ratios of these molecules are not shown.

AmB-loaded liposomes (AmB-LLs) penetrate more efficiently into various organs (7, 8), penetrate the cell wall (9), and show reduced toxicity at higher, more effective doses of AmB than the second most commonly used AmB product, deoxycholate detergent-solubilized AmB (5, 6, 10, 11). AmB is an amphipathic molecule. Its long lipophilic polyene end intercalates into the lipid bilayer of liposomes, while its hydrophilic end is positioned on the liposomal surface as modeled in Fig. 1. Commercial untargeted spherical AmB-LLs are called AmBisome (12, 13). However, AmB-LLs still produce AmB human toxicity, such as renal toxicity in 50% of patients (5, 6, 11). When infected mice are treated with AmB-LLs, viable numbers of A. fumigatus cells in homogenized lung tissue are reduced by only 70% (14, 15), leaving large fungal cell populations behind. This large residual fungal population may result in recurrence and subsequent mortality after treatment. We explored the targeting of AmB-LLs to Aspergillus fumigatus cells to meet the pressing need to improve the quality of antifungal drug formulations (1).

10.1128/mSphere.00025-19.5 TABLE S1 Liposome compositions. Comparison of the chemical composition of liposomes discussed in the article. Download Table S1, TIF file, 0.4 MB. Copyright © 2019 Ambati et al. 2019 Ambati et al. This content is distributed under the terms of the Creative Commons Attribution 4.0 International license.

Liposomes biochemically resemble endogenous exosomes (16–18). They efficiently penetrate the endothelial barrier and reach target cells deep in most major organs for the “passive delivery” of variously loaded therapeutic drugs (19–22). Targeted liposomes have binding specificity for a plasma membrane antigen to enable the “active delivery” of a packaged therapeutic to diseased cells. Targeting is most commonly achieved with a monoclonal antibody such that immunoliposomes bind a specific cell type or types. Over 100 publications, most focused on particular types of cancer cells, show that targeted immunoliposomes improve the cell type specificity of drug delivery and reduce toxicity. Therapeutic drug-loaded immunoliposomes include those targeting cells expressing vascular endothelial growth factor receptors (VEGF-Rs) 2 and 3 (23), the oxytocin receptor (24), the epidermal growth factor receptor (EGFR) (25), CD4 (26), and HER2 (27, 28). The active delivery of immunoliposomes generally improves cell type specificity and drug effectiveness 3- to 10-fold (24, 29, 30) over those with passive delivery. A wide variety of drugs have been delivered via targeted liposomes, including toxins such as doxorubicin, paclitaxel, and rapamycin (31, 32), growth hormones such as transforming growth factor β (33), and analgesics such as the indomethacin (24). We are unaware of any reports of immunoliposomes specifically targeting antifungals to invasive fungal cells; however, the immunotargeting of AmB-loaded liposomes to the vessel wall of pulmonary capillary cells in A. fumigatus-infected mouse lungs results in increased mouse survival rates (15).

Dectin-1 is a transmembrane receptor expressed in natural killer lymphocytes encoded by the *CLEC7A* (C-type lectin domain containing 7A, beta-glucan receptor) gene in mice and humans. Dectin-1 binds various beta-glucans in fungal cell walls and is the primary receptor for transmembrane signaling of the presence of cell wall components from the surface of fungal cells, stimulating an innate immune response (34–37). Human and mouse Dectin-1 proteins are 244- and 247-amino-acid (aa)-long plasma membrane proteins, respectively, although there are mRNA splice variants producing shorter human isoforms. Dectin-1 floats in the membrane as a monomer but binds to beta-glucans as a dimer, as modeled in our design of Dectin-1-targeted liposomes shown in Fig. 1 (38). The 176-amino-acid-long (20-kDa) extracellular C-terminal beta-glucan binding domain is often manipulated alone as sDectin-1 (soluble dectin-1; DEC). The beta 1→3 glucans are a structurally diverse class of polysaccharides, and consequently, sDectin-1 binds various model beta-glucans differentially, with 50% inhibitory concentrations (IC_50_s) ranging from 2.6 mM to 2.2 pM (37). sDectin-1 is reported to recognize A. fumigatus cell wall components much more efficiently on germinating conidia and germ tubes than on dormant conidia or mature hyphae (39, 40). Having pan-fungal binding activity, Dectin-1 may provide broader antifungal targeting abilities for liposomes than a monoclonal antibody (41).

The goal of our research has been to develop a targeted liposomal strategy that improves antifungal drug delivery and enhances therapeutic efficacy. To begin to address this goal, we tested the hypothesis that AmB-LLs targeted directly to beta-glucans in the cell wall of A. fumigatus by sDectin-1 will have enhanced antifungal activity over current untargeted AmB-LLs.

(This article was submitted to an online preprint archive [42].)

## RESULTS

### Preparation of AmB-loaded sDectin-1-coated liposomes.

Pegylated liposomes were remotely loaded with 11 mol% AmB relative to moles of liposomal lipids to make control AmB-LLs, which are similar in structure and AmB concentration to commercial unpegylated AmBisome preparations (see Materials and Methods; see also Table S1 in the supplemental material). sDectin-1 (DEC) (Fig. S1 and S2) and bovine serum albumin (BSA) were coupled to a pegylated lipid carrier, DSPE-PEG. One mole percent DSPE-PEG-DEC was incorporated into AmB-LLs to make sDectin-1-coated DEC-AmB-LLs (Fig. 1), and 0.33 mol% DSPE-PEG-BSA was incorporated into AmB-LLs to make BSA-AmB-LLs. This mole ratio of 22-kDa sDectin-1 and 65-kDa BSA results in equivalent microgram amounts of protein coating each set of liposomes. Because these protein-coated liposomes were made from the same AmB-LLs, all three liposomal preparations contain 11 mol% AmB relative to moles of lipid. Two moles percent of DHPE (dihexadecanoyl-glycero-phosphoethanolamine)-rhodamine was loaded into all three classes of liposome (Fig. 1).

10.1128/mSphere.00025-19.1 FIG S1 The modified mouse sDectin-1 DNA *MmsDectin1lyshis* and protein MmsDectin-1. (A) The codon-optimized DNA sequence of *MmsDECTIN1lyshis* was cloned into pET-45B (NCBI BankIT submission 2173810; length, 577 bp). The vector pET-45b sequence is highlighted in red, with the start codon underlined. Cloning sites are in green, codons for Gly and Ser (G and S) flexible linker residues are in yellow, reactive Lys (K) residues are in purple, mouse sDecetin-1 is in light blue, the terminal Ala codon to put stop codons in frame is in yellow, and stop codons are in bold. (B) The modified mouse sDectin-1 protein being synthesized. The N terminus and His tag from the pET-45B vector are in red, Gly and Ser flexible linker residues are in yellow, reactive Lys residues are in purple, and mouse sDecetin-1 is in light blue. The final Ala residue/codon is to put stop codons and PacI site in frame. Length, 199 amino acids; molecular weight, 22,389.66 g/mol; theoretical pI, 7.74. Download FIG S1, TIF file, 0.10 MB. Copyright © 2019 Ambati et al. 2019 Ambati et al. This content is distributed under the terms of the Creative Commons Attribution 4.0 International license.

10.1128/mSphere.00025-19.2 FIG S2 SDS-PAGE analysis of sDectin-1 in cell extracts and after affinity purification. sDectin-1 protein was produced in the BL21 strain of E. coli grown in Luria broth overnight from the pET-45B plasmid without IPTG induction. The protein was solubilized in GuHCl buffers, purified by nickel-nitrilotriacetic acid (Ni-NTA) resin, and examined by SDS-PAGE after GuHCl was removed by dialysis. Extraction of protein into buffers that also contained reducing agent 2-mercaptoethanol and Triton X-100 detergent greatly increased recovery from insoluble inclusion bodies (center lanes) relative to buffers without them (right lanes). Protein was examined on a 12% acrylamide gel stained with Coomassie blue. The approximate molecular weight of modified sDectin-1 (22 kDa) is indicated. Extraction of these cells with urea buffers even at 60°C yielded very little protein (not shown). Download FIG S2, TIF file, 0.3 MB. Copyright © 2019 Ambati et al. 2019 Ambati et al. This content is distributed under the terms of the Creative Commons Attribution 4.0 International license.

### DEC-AmB-LLs bind strongly to fungal cells.

**FIG 2:**
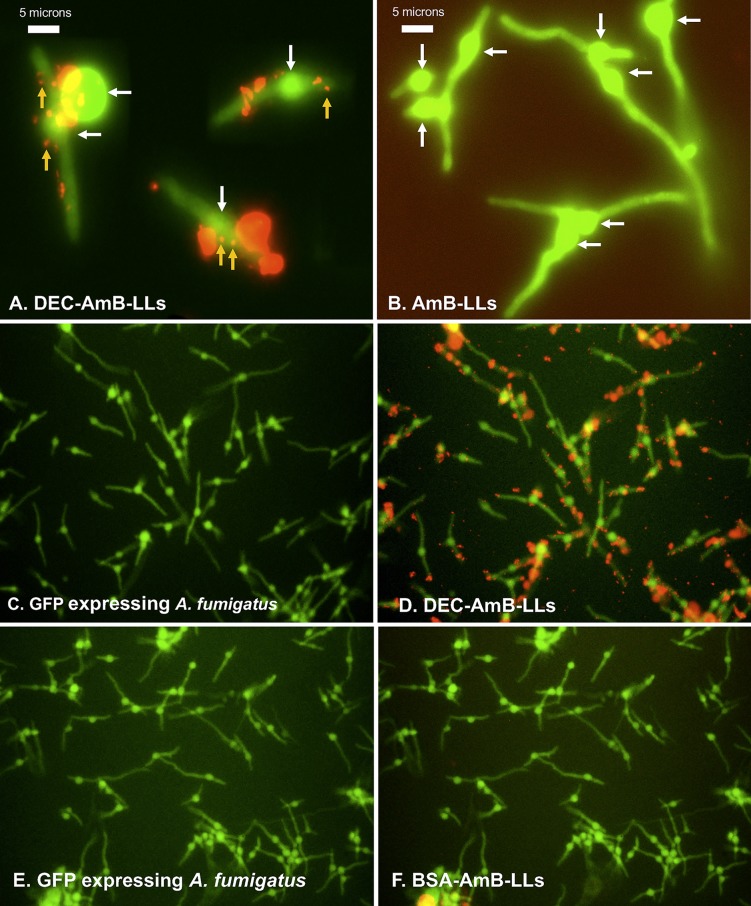
sDectin-1-coated DEC-AmB-LLs bound strongly to germinating conidia and germ tubes of A. fumigatus, while AmB-LLs and BSA-AmB-LLs did not. A. fumigatus conidia were germinated and grown for 8 to 10 h in VMM plus 1% glucose at 37°C in 24-well microtiter plates before being fixed and stained with fluorescent liposomes. (A) Rhodamine red fluorescent DEC-AmB-LLs bound swollen conidia (white arrows) and germ tubes of A. fumigatus. (B) Rhodamine red fluorescent AmB-LLs did not bind at detectable frequencies. No AmB-LLs were detected even when the red channel was enhanced as in this image. The smallest red dots in plate A represent individual 100-nm-diameter liposomes viewed based on their fluorescence (orange arrows). Large clusters of liposomes form the more brightly red stained areas. (C and D) Staining with DEC-AmB-LLs. (E and F) Staining with BSA-AmB-LLs. Conidia in panels C and E were photographed in the green fluroescent channel, while for those in panels D and F, green and red fluorescent channels were combined. Labeling was performed in LDB1 for 60 min. All three liposome preparations were diluted 1:100 such that liposomal sDectin-1 and BSA proteins were at final concentrations of 1 μg/100 μl. Germlings were viewed in the green channel alone for cytoplasmic fluorescent EGFP expression and in the red channel for rhodamine fluorescent liposomes. Cells in panels A and B were photographed at ×63 magnification under oil immersion in a compound fluorescence microscope, and red fluorescence was further enhanced in panel B to detect potentially individual liposomes. Cells in panels C through F were photographed at ×20 on an inverted fluorescence microscope.

In assays performed on A. fumigatus germlings, rhodamine red fluorescent DEC-AmB-LLs bound strongly to germinating conidia and to germ tubes, as shown in Fig. 2. The sDectin-1-targeted liposomes often bound in large numbers that formed aggregates at particular regions. While 100-nm liposomes are too small to be resolved by light microscopy, individual liposomes were visible as somewhat uniformly sized small red fluorescent dots (orange arrows in Fig. 2A), which are easily detected due to their each containing an estimated 3,000 rhodamine molecules (Fig. 1). From examinations of larger fields of germlings it appears that essentially all germlings bind DEC-AmB-LLs (Fig. 2C and D). AmBisome-like AmB-LLs (Fig. 2B) and BSA-coated liposomes (BSA-AmB-LLs) (Fig. 2E and F) did not bind detectably to germinating conidia or germ tubes when tested at the same concentration. Maximum labeling by DEC-AmB-LLs was achieved within 15 to 30 min and the strong red fluorescent signals of DEC-AmB-LLs bound to cells were maintained for weeks when fixed cells were stored in the dark in phosphate-buffered saline (PBS) at 4°C.

**FIG 3:**
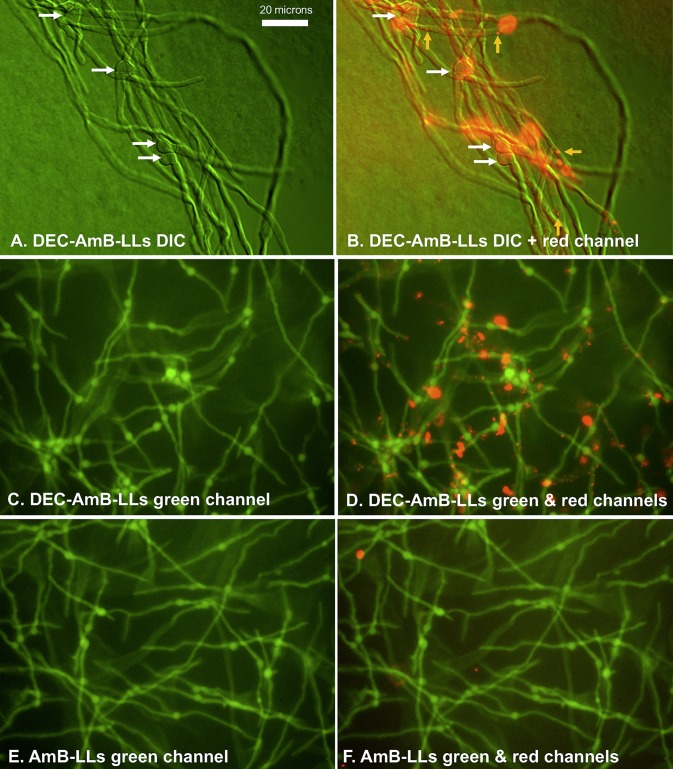
sDectin-1-coated DEC-AmB-LLs bound germinating conidia and hyphae of mature A. fumigatus cells, while untargeted AmB-LLs and BSA-AmB-LLs did not. A. fumigatus conidia were germinated and grown for 16 h in VMM plus 1% glucose at 37°C in 24-well microtiter plates before being fixed and stained with fluorescent liposomes. Cells were stained with rhodamine red fluorescent DEC-AmB-LLs diluted 1:100 such that sDectin-1 was at 1 μg/100 μl (A to D) and with the equivalent amount of red fluorescent AmB-LLs for 60 min (E and F). (A) DIC image alone. (B) Combined DIC and red fluorescence image. Panels A and B show that rhodamine fluorescent DEC-AmB-LLs bound to germinating conidia (white arrows) and hyphae. In panel B, the smallest red dots represent individual 100-nm liposomes (orange arrows). (C to F) Cytoplasmic EGFP and the red fluorescence of liposomes. Panels C and D show that nearly all conidia and most hyphae stained with DEC-AmB-LLs. Panels E and F show that AmB-LLs did not bind. Cells in panels A and B were photographed at ×63 under oil immersion, and those in panels C to F were photographed at ×20 on an inverted fluorescence microscope.

DEC-AmB-LLs also bound to germinating conidia and most hyphae from more mature cultures, as shown in Fig. 3. Again, the sDectin-1-targeted liposomes often bound in aggregates, but some fairly uniformly sized individual small red dots are visible (orange arrows in Fig. 3A), which appear to be individual fluorescent liposomes. AmB-LLs did not bind significantly to older conidia or mature hyphae (Fig. 3E and F), nor did BSA-AmB-LLs.

**FIG 4:**
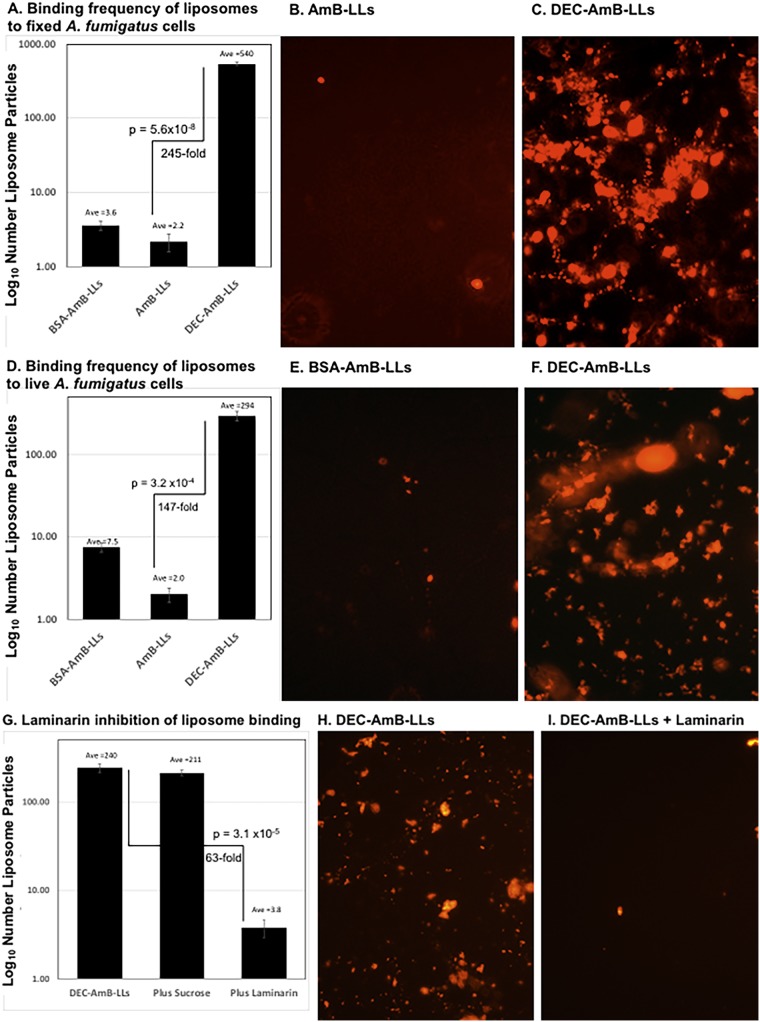
sDectin-1-coated DEC-AmB-LLs bound 2 orders of magnitude more frequently to A. fumigatus than control AmB-LLs, and binding was inhibited by soluble beta-glucan. Samples of 4,500 A. fumigatus conidia were germinated and grown at 37°C for 36 h in VMM plus 1% glucose, fixed in formalin or examined live, and incubated for 1 h with a 1:50 dilution of liposomes in liposome dilution buffer LDB1. Unbound liposomes were washed out. Multiple fields of red fluorescent images were photographed at ×20 and red fluorescence enhanced equivalently for all images. Each photographic field contained approximately 25 swollen conidia and an extensive network of hyphae (not shown). (A, B, and C) Labeling of formalin-fixed cells. (D, E, and F) Labeling of live cells. (G, H, and I) Inhibition of DEC-AmB-LL labeling of fixed cells by 1 mg/ml of laminarin, a soluble beta-glucan versus 1 mg/ml of sucrose as a control. (A, D, and G) The numbers of red fluorescent liposomes and clusters of liposomes were counted, averaged per field, and plotted on a log_10_ scale. The numerical average is indicated above each bar and on the vertical axis. Standard errors are shown. Fold differences and *P* values are indicated for the performance of DEC-AmB-LLs relative to AmB-LLs. Examples of photographic fields of liposomes used to construct the adjacent bar graphs are shown in panels B, C, E, F, H, and I.

On plates covered with dense layers of mature hyphae, the numbers of bound liposomes and liposome aggregates were counted in multiple fluorescent images. DEC-AmB-LLs bound to both formalin-fixed (Fig. 4A to C) and live (Fig. 4D to F) A. fumigatus cells 100- to 200-fold more efficiently than AmB-LLs or BSA-AmB-LLs. Labeling by DEC-AmB-LLs was inhibited 50-fold by the inclusion of soluble beta-glucan and laminarin but not sucrose, confirming that binding was beta-glucan specific (Fig. 4G to I). Finally, DEC-AmB-LLs labeled Cryptococcus neoformans cells and Candida albicans pseudohyphae (Fig. S3), while control liposomes did not. However, the frequency of DEC-AmB-LL binding to C. albicans pseudohyphae was very low. In short, Dectin-coated AmB-loaded liposomes bound efficiently to a variety of fungal cells, while control liposomes did not.

10.1128/mSphere.00025-19.3 FIG S3 sDectin-coated liposomes, DEC-AmB-LLs, bound strongly to Candida albicans and Cryptococcus neoformans cells. (A, C, and E) Bright-field images of C. albicans strain Sc5314 and C. neoformans strain H99 labeled with DEC-AmB-LLs diluted 1:100 in LDB1; (B, D, and F) combined bright-field and red fluorescence images showing that rhodamine red fluorescent DEC-AmB-LLs bound strongly to these cells. Plain uncoated AmB-LLs and BSA-AmB-LLs did not bind detectably to these cells (not shown). Cells in panels A and B were photographed at ×63 under oil immersion, and those in panels C to F at ×20 on an inverted fluorescent microscope. Download FIG S3, TIF file, 0.8 MB. Copyright © 2019 Ambati et al. 2019 Ambati et al. This content is distributed under the terms of the Creative Commons Attribution 4.0 International license.

### Killing and growth inhibition of fungi by DEC-AmB-LLs.

**FIG 5:**
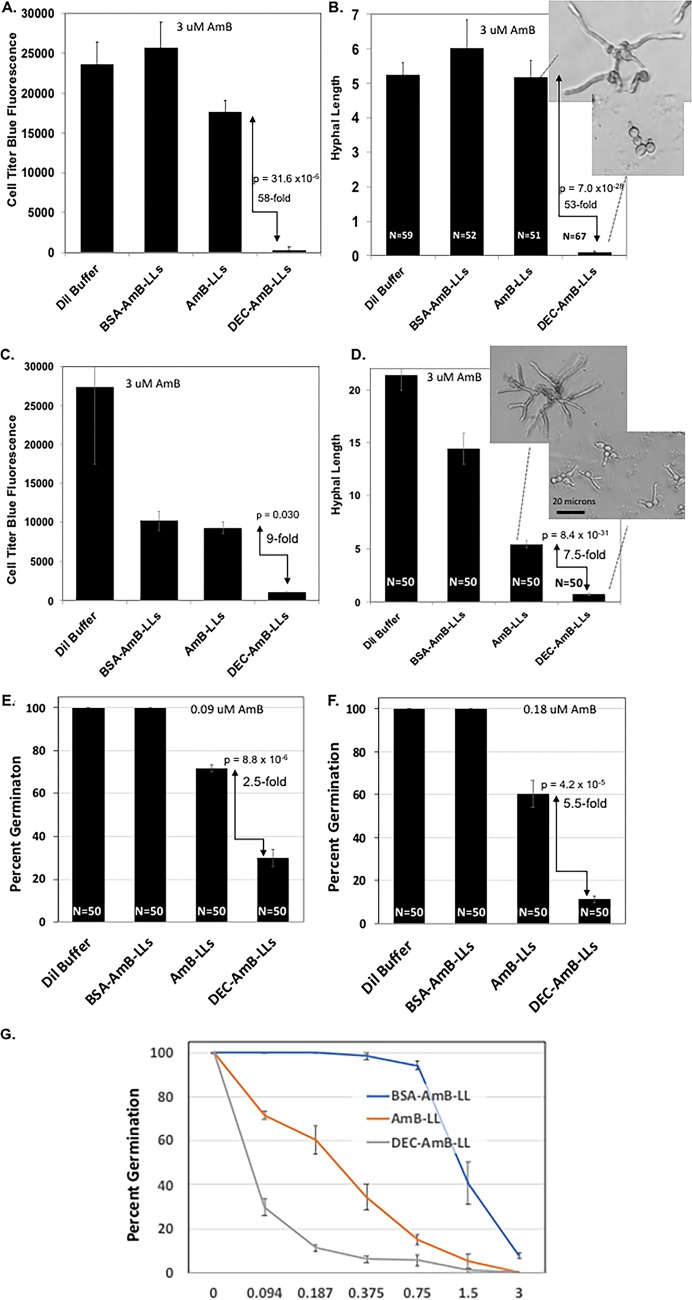
DEC-AmB-LLs inhibited the growth A. fumigatus far more efficiently than AmB-LLs. Samples of 4,500 A. fumigatus conidia were germinated and grown in 96-well microtiter plates in VMM plus 1% glucose for 8 to 56 h at 37°C and treated at the same time with liposome preparations delivering the indicated concentrations of AmB to the growth media—for panels A through D, 3 μM AmB, a 1:300 fold dilution of all three liposome preparations; for panel E, 0.09 μM; for panel F, 0.18 μM; and for panel G, 0.9 to 3 μM)—or an equivalent amount of liposome dilution (Dil) buffer LDB2. Viability and growth were estimated using CellTiter-Blue reagent (A and C) or by measuring hyphal length (B and D) or by scoring percent germination (E, F, and G). Background fluorescence from wells with CellTiter-Blue reagent in the media but lacking cells and liposomes was subtracted. Standard errors are indicated. Fold differences and *P* values are indicated for comparisons of the performance of DEC-AmB-LLs to AmB-LLs. Inset photos in panels B and D show examples of the length of hyphae assayed for AmB-LL- and DEC-AmB-LL-treated samples. One unit of hyphal length in panels B and D equals 5 μm. Panels A and B and panel C and D compare the results from two biological replicate experiments with independently conjugated sDectin-1 and assembled liposomes.

We performed various fungal cell growth and viability assays after treating A. fumigatus with liposomes delivering AmB concentrations near its estimated 50% effective dose (ED_50_) of 2 to 3 μM AmB (43) or below its estimated MIC of 0.5 μM for various strains of A. fumigatus (44). In most of these experiments, 4,500 conidia were germinated and incubated for 12 to 72 h in 96-well microtiter plates along with drug-loaded liposomes. Long incubation times were often needed to resolve differences among the liposome preparation delivering higher concentrations of AmB. Figure 5 shows that targeted DEC-AmB-LLs killed or inhibited the growth of A. fumigatus cells far more efficiently than BSA-AmB-LLs or uncoated AmB-LLs delivering the same concentrations of AmB. Assays with CellTiter-Blue reagent, which assesses cytoplasmic reductase activity as a proxy for cell integrity and viability, showed that treating cells with DEC-AmB-LLs delivering 3 μM AmB killed A. fumigatus more than an order of magnitude more effectively than AmBisome-like AmB-LLs or BSA-AmB-LLs (Fig. 5A). As a second method to score liposomal AmB activity, we measured hyphal length. Hyphal-length assays gave a similar result, showing that DEC-AmB-LLs delivering 3 μM AmB were far more effective at inhibiting hyphal growth than AmB-LLs or BSA-AmB-LLs (Fig. 5B). To confirm that DEC-AmB-LLs were still functional in an alternate sDectin-1 renaturation buffer, we executed a complete biological replicate experiment, wherein sDectin-1 and liposomes were prepared in RN#8 buffer instead of RN#5 (see Materials and Methods). We obtained a similar although less dramatic result with these liposomes delivering 3 μM AmB (Fig. 5C and D).

A third assay of liposomal AmB activity was employed, which measured the percentage of conidia that germinated in the presence of the various liposomal preparations (Fig. 5E and F). DEC-AmB-LLs delivering as little as 0.09 μM and 0.187 μM AmB inhibited the germination of A. fumigatus conidia significantly better than AmB-LLs or BSA-AmB-LL. The dose response to DEC-AmB-LLs based on a germination assay performed after a fixed period of growth using AmB concentrations from 0.09 to 3 μM is shown in Fig. 5G. DEC-AmB-LLs outperformed AmB-LLs and BSA-AmB-LLs over a wide range of concentrations.

### Reduced animal cell toxicity of DEC-AmB-LLs.

AmB-LLs and AmB deoxycholate micelles were slightly more toxic to HEK293 human embryonic kidney cells than DEC-AmB-LLs or BSA-AmB-LLs based on CellTiter-Blue assays of cell viability (Fig. S4).

10.1128/mSphere.00025-19.4 FIG S4 sDectin-1-coated DEC-AmB-LLs and BSA coated BSA-AmB-LLs were less toxic to HEK293 cells than uncoated AmB-LLs. Human embryonic kidney HEK293 cells grown to 30 to 40% cell density in RPMI lacking red indicator dye in 96-well microtiter plates. Cells were treated for 2 h with the AmB-loaded liposomes indicated or a deoxycholate micelle suspension of AmB (DOC), washed twice, and then incubated for an additional 16 h. All treatments delivered a final concentration of 30 or 15 μM AmB into the media. The 0 μM control wells received an amount of liposome dilution buffer LDB2 equivalent to the 30 μM treatment. CellTiter-Blue assays estimated cell viability and survival. Background fluorescence from wells with CellTiter-Blue reagent in the media but lacking cells and liposomes was subtracted. Standard errors are indicated. Percent difference and *P* values are indicated for comparisons of the performance of DEC-AmB-LLs to AmB-LLs. Download FIG S4, TIF file, 0.1 MB. Copyright © 2019 Ambati et al. 2019 Ambati et al. This content is distributed under the terms of the Creative Commons Attribution 4.0 International license.

## DISCUSSION

We demonstrated that sDectin-1-targeted, AmB-loaded DEC-AmB-LLs are significantly more effective at binding to and inhibiting the growth of fungal cells and are slightly less toxic to human cells than uncoated AmB-LLs. The biochemical manipulation of mouse or human sDectin-1 has been complicated because the proteins easily aggregate and become insoluble and inactive in aqueous buffers. The problem of protein aggregation and insolubility may partly be due to their composition, due to the presence of 11% to 13% hydrophobic amino acids and three disulfide cross-links in their carbohydrate recognition domains. A wide variety of protein chemical manipulations have been applied to improve sDectin-1 solubility, with mixed success. For example, the solubility and utility of sDectin-1 were increased by tethering it to the 56-amino-acid-long B1 domain of streptococcal protein G (45) or, more commonly, to the 232-amino-acid-long Fc constant region of IgG1 antibody (40). Bacterially produced, nonglycosylated sDectin-1 renatured from inclusion bodies and deglycosylated and native mammalian cell-produced sDectin-1 all retain indistinguishable beta-glucan binding activity (38, 46, 47). Therefore, we proceeded with sDectin-1 production in Escherichia coli, with the potential for highest yield and lowest cost. We overcame sDectin-1’s solubility problems by combining a variety of old and new approaches, including (i) the use of a very short charged peptide tag LysHisLys; (ii) the inclusion of 6 M guanidine hydrochloride (GuHCl) during protein extraction, purification, and chemical modification; (iii) the inclusion of the protein solubilizing agent, arginine, during renaturation, liposomal loading, and storage; and (iv) the inclusion of a sulfhydryl-reducing agent, 2-mercaptoethanol, in all steps. sDectin-1 is reported to bind efficiently to A. fumigatus germinating conidia and germ tubes but inefficiently, if at all, to mature hyphae and not at all to ungerminated conidia (39, 40, 48). Our data with sDectin-1-coated AmB-loaded liposomes are partially consistent with previous observations, except that we observed reasonably efficient staining of mature hyphae (Fig. 3 and 4). Poor cell or hyphal binding may be explained by polymorphic expression of beta-glucans in different stages of fungal cell growth (48, 49). During infection, however, interaction with host immune cells reportedly exposes otherwise masked beta-glucans (50), with the potential to enhance the potency of the targeted DEC-AmB-LLs. In this study, sDectin-1-coated fluorescent DEC-AmB-LLs bound efficiently to germinating conidia, germ tubes, and hyphae, suggesting that our modified sDectin-1 presented on the surface of liposomes retained its ability to form complexes with affinity for fungal beta-glucans expressed at various stages of growth. Our data showed for the first time that *in vitro* chemically modified sDectin-1 (DSPE-PEG-DEC) retained its fungal cell binding specificity. Furthermore, DEC-AmB-LL binding was rapid and DEC-AmB-LLs remained stably bound to cells for weeks. Perhaps the greater avidity of liposome coated with ∼1,500 sDectin-1 molecules ensured the rapid efficient binding and very slow release of bound liposomes, parallel to the avidity of pentameric IgM antibody. The presence of thousands of rhodamine molecules on each liposome (Fig. 1) should have greatly increased the chance of detecting unambiguous fluorescent signals relative to detecting the binding of sDectin-1 dimers, as reported in previous studies. DEC-AmB-LLs bound very efficiently to both live and formalin-fixed hyphae. Side-by-side comparisons are needed to determine if formalin fixation improved binding or blocked access to beta-glucans.

In a large number of experiments using different binding buffers including BSA and various incubation periods, we never detected any significant affinity of uncoated AmB-LLs or BSA-AmB-LLs for fungal cells, with one exception. In preliminary experiments in which BSA was omitted during the incubation, we observed that BSA-AmB-LLs bound modestly well to A. fumigatus germinated conidia, while we still did not observe AmB-LL binding. In contrast, Chavan et al. (51) detected efficient binding of fluorescent pegylated liposomes to primary tips and septa on A. fumigatus hyphae even in the presence of serum (51). We cannot account for this disparity between their data and ours, except that their liposomes had a different lipid composition and lacked both sDectin-1 and AmB (Table S1).

*Aspergillus*, *Candida*, and *Cryptococcus* species belong to three evolutionarily disparate groups of fungi—the Hemiascomycetes, Euascomycetes, and Hymenomycetes, respectively—and are separated from common ancestry by hundreds of millions of years (52). DEC-AmB-LLs bound specifically to all three. This suggests that the beta-glucans found in the outer cell wall of many pathogenic fungi will be conserved enough in structure to bind sDectin-1-targeted liposomes if they are accessible. However, beta-glucan expression and masking from host detection may vary widely among fungal species (48, 49, 53). Our assays also showed inefficient binding of DEC-AmB-LLs to C. albicans, consistent with the masking of beta-glucans reported for this species (54–56). We did not test for binding to zygomycete pathogens, but a targeted liposome approach may also increase drug efficacy for at least some members of the Mucorales. For example, beta-glucans in the Rhizopus oryzae cell wall induce a Dectin-1-dependent response (57).

In various biological and experimental replicate experiments using different assay methods, we showed that DEC-AmB-LLs killed or inhibited A. fumigatus cells far more efficiently than AmBisome-like AmB-LLs delivering the same level of AmB. In all of our experiments, DEC-AmB-LLs were severalfold to more than an order of magnitude more fungicidal than control liposomes over a wide variety of AmB concentrations tested that were near or below the estimated ED_50_ of 3 μM. We detected significantly stronger activity of DEC-AmB-LLs over AmB-LLs even at AmB concentrations as low as 0.094 μM AmB, well below AmB’s MIC. DEC-AmB-LLs significantly decreased the amount of AmB required for an ED_50_ or a MIC for A. fumigatus. The time of incubation with drug-loaded liposomes strongly influences the ability to resolve differences among the three liposome preparations. For example, when all three liposome preparations delivered high AmB concentrations (e.g., 0.75, 1.5, and 3 μM), they caused a lag in the germination of conidia. Hence, longer incubation periods were needed to allow sufficient fungal growth to resolve the improved performance of DEC-AmB-LLs. Short incubation periods were needed to resolve differences at low AmB concentrations (e.g., 0.37, 0.18, and 0.94 μM). Thus, we were unable to obtain a dose-response curve that reflected the optimal performance of DEC-AmB-LLs over a wide range of AmB concentrations. Finally, AmB-containing liposomes were added to plates at the same time as conidia during the growth inhibition assays. Thus, the CellTiter-Blue and hyphal-length assays combined inhibition of germination, germling growth, and hyphal extension but may be skewed toward inhibition of early stages of growth. Future inhibition studies are needed that focus just on hyphal stages to make this work even more relevant to a clinical setting, but our observation that older hyphae are bound by DEC-AmB-LLs suggests that improved killing of mycelia is likely.

Looking forward, there are a number of important variables we have not yet explored. For example, we coated liposomes with a single concentration of sDectin-1, approximately 1,500 molecules per liposome, and do not know if this is the optimal concentration for cell binding and drug delivery. Also, although DEC-AmB-LLs were superior in all aspects to AmB-LLs, we do not know the ratio of growth inhibition to killing at different AmB concentrations. Efficient killing can be followed by rapid outgrowth of the remaining cells, obscuring the results. This is particularly relevant to the treatment of aspergillosis, because current drug formulations only partially reduce the fungal cell load in mice and humans. An important next step in our research needs be an examination of the performance sDectin-1-coated antifungal drug-loaded liposomes in animal models of fungal diseases. Only one human mutation in Dectin-1 Y238Term, very close to its natural termination codon 245, has been reported at the Human Gene Mutation Database (http://www.hgmd.cf.ac.uk/ac/index.php). Hence, the human sDectin-1 sequence itself may not present problems of immunogenicity in a clinical setting. However, we do not know if the design of liposomal sDectin-1 that included a LysHisLys peptide tag for lysine coupling to a lipid carrier will be immunogenic.

In summary, sDectin-1 polypeptides conjugated to a pegylated lipid carrier and inserted as monomers into liposomes must float together to form functional dimers or multimers as they bind beta-glucans, or we would not have observed the strong efficient binding of DEC-AmB-LLs to fungal cells. Our DEC-AmB-LLs efficiently bind beta-glucans in the cell walls of diverse fungal species. Multiple growth and viability assays on DEC-AmB-LLs delivering AmB concentrations from 0.094 to 3 μM suggest that sDectin-1-coated liposomes greatly improved the performance of liposomal AmB. Taking these results together, it is reasonable to propose that sDectin-1-coated liposomes have significant potential as pan-fungal carriers for targeting antifungal therapeutics.

## MATERIALS AND METHODS

### Fungal growth.

Aspergillus fumigatus strain A1163 was transformed with plasmid pBV126 (described by Khang et al. [58]) carrying enhanced green fluorescent protein (EGFP) under the control of Magnaporthe oryzae ribosomal protein 27 promoter to make strain AEK012. AEK012 was used to monitor fungal cells in some experiments. A. fumigatus spores were inoculated on petri plates containing Vogel’s minimal medium (VMM; 1% glucose, 1.5% agar) and grown for 7 days, at which time conidia were collected in PBS plus 0.1% Tween. For fluorescent liposome localization and for growth inhibition and killing assays, 20,000 and 4,500 AEK012 conidia were plated on 24-well and 96-well plates, respectively, in VMM, 1% glucose, and 0.5% BSA at 37°C for various periods ranging from 8 h to 56 h (59, 60). Candida albicans Sc5314 and Cryptococcus neoformans H99 were pregrown in yeast extract-peptone-dextrose (YPD) liquid medium overnight. The cells were then washed 3 times with sterile water, resuspended in VMM, and grown at 35°C for 10 h. All fungal cell growth was carried out in a biosafety level 2 (BSL2) laboratory. Prior to liposomal staining, most fungal preparations were washed 3 times with PBS, fixed in 4% formaldehyde in PBS for 15 to 60 min, washed twice, and stored at 4°C in PBS.

### Production of soluble Dectin-1.

The sequence of the codon-optimized E. coli expression construct with MmsDectin-1lyshis (NCBI BankIT #2173810) cloned into pET-45B (GenScript) is shown in Fig. S1. The sequence encodes a slightly modified 198-aa-long sDectin-1 protein containing a vector specified N-terminal (His)_6_ affinity tag, a flexible GlySer spacer, two lysine residues, and another flexible spacer followed by the C-terminal 176-aa-long murine sDectin-1 domain. E. coli strain BL21 containing the MmsDectin-1-pET45B plasmid was grown overnight in 1 liter of Luria broth without isopropyl-β-d-thiogalactopyranoside (IPTG) induction (Fig. S2). Modified sDectin-1 was extracted from cell pellets in buffer containing 6 M GuHCl (pH 8.0; Fisher BioReagents; BP178), 0.1 M Na_2_HPO_4_:NaH_2_PO_4_ (1:9), 10 mM triethanolamine, 100 mM NaCl, 5 mM 2-mercaptoethanol, and 0.1% Triton X-100, which was modified from a GuHCl buffer used an earlier study (61). sDectin-1 was bound to a nickel affinity resin (Qiagen; 30210) in the same buffer, washed in the same adjusted to pH 6.3, and eluted in this buffer adjusted to pH 4.5. The pH of the eluted protein was immediately neutralized to pH 7.2 with 1 M pH 10.0 M triethanolamine for long-term storage. Forty milligrams of >95% pure protein was recovered per liter of Luria broth (Fig. S2). Samples of sDectin-1 at 6 μg/μl in the same GuHCl buffer with freshly added 5 mM 2-mercaptoethanol were adjusted to pH 8.3 with 1 M triethanolamine (pH 10) and reacted with a 4-molar excess of DSPE-PEG-3400-NHS (Nanosoft Polymers; 1544-3400) for 1 h at 23°C to make DSPE-PEG-DEC. Gel exclusion chromatography on Bio-Gel P-6 acrylamide resin (Bio-Rad; 150-0740) in renaturation and storage buffer RN#5 (0.1 M NaH_2_PO_4_, 10 mM triethanolamine [pH 8.0], 1 M l-arginine, 100 mM NaCl, 5 mM EDTA, 5 mM 2-mercaptoethanol) removed unincorporated DSPE-PEG and GuHCl (45, 62). DSPE-PEG-BSA was prepared from BSA (Sigma; A-8022) by the same protocol, minus the GuHCl from DSPE-PEG labeling buffers and l-arginine from RN#5 buffer.

### Remote loading of AmB, sDectin-1, BSA, and rhodamine into liposomes.

Sterile pegylated liposomes were obtained from FormuMax Sci. Inc. (DSPC–CHOL–mPEG2000-DSPE, 53:47:5 mole ratio, 100-nm diameter, 60 μmol/ml lipid in a liposomal suspension, FormuMax F10203A). Small batches of liposomes were remotely loaded with 11 mol% AmB (Sigma; A4888) relative to 100% liposomal lipid to make AmBisome-like AmB-LLs used throughout this study. For example, AmB (2.8 mg, 3 μmol, or 20 mol%) was dissolved in 13 μl of dimethyl sulfoxide (DMSO) by heating for 10 to 20 min with occasional mixing at 60°C to make an oil-like clear brown AmB solution. Two hundred fifty microliters of sterile liposomal suspension (15 μmol of liposomal lipid in 50% liposome suspension) was added to the AmB oil and mixed on a rotating platform for 3 days at 37°C, followed by centrifugation for 10 min at 100 × *g* to pellet the AmB oil. Dissolving this oil phase in 0.5 ml of DMSO and spectrophotometry at A407 relative to AmB standards in DMSO showed that 1.3 μmol of AmB remained undissolved and 1.7 μmol of AmB (11 mol%) were retained in liposomes. Subsequent gel exclusion chromatography of loaded liposomes over a 10-ml BioGel A-0.5 M agarose resin (Bio-Rad; 151-0140) revealed no detectable AmB in the salt volume, and essentially all of the AmB was retained by liposomes. Longer incubations resulted in higher percentages of AmB in liposomes. Commercial AmBisome (Gilead) liposomes are not pegylated and contain 10.6 mol% AmB relative to lipid (Table S1).

The DSPE-PEG-sDectin-1 and DSPE-PEG-BSA conjugates in RN#5 buffer and PBS, respectively, were integrated via their DSPE moiety into the phospholipid bilayer membrane of AmB-LLs at 1.0 and 0.33 mol% of protein relative to moles of liposomal lipid by 30 to 60 min of incubation at 60°C to make DEC-AmB-LLs and BSA-AmB-LLs. We had previously determined that both arginine and guanidine prevented sDectin-1 aggregation and were aids to functional renaturation, as shown for other proteins (63–65). However, the arginine in RN#5 is a reported inhibitor of *Aspergillus* growth (66). Therefore, we examined a second method of preparing liposomes in which the newly coupled DSPE-PEG-sDectin-1 was exchanged into renaturation buffer RN#8 (50 mM carbonate [pH 8.5], 0.5 M guanidine hydrochloride, 250 mM NaCl, 2 mM 2-mercaptoethanol, 0.1% Triton, 20% glycerol) and loaded into liposomes in RN#8. We concluded that DSPE-PEG-sDectin-1 and liposomes prepared using in RN#5 were slightly superior at labeling fungal cells to those prepared in RN#8. During the same 60°C incubation, the red fluorescent tag, Lissamine rhodamine B-DHPE (Invitrogen, #L1392) was also incorporated at 2 mol% relative to liposomal lipid into sDectin-1- and BSA-coated liposomes and AmB-LLs (67–69). Gel exclusion chromatography on BioGel A-0.5 M resin confirmed that rhodamine and protein insertions into liposomes were essentially quantitative. DEC-AmB-LLs stored at 4°C in RN#5 retained fungal cell binding specificity for 2 months.

### Microscopy of liposome binding.

Formalin-fixed or live fungal cells were incubated with liposomes in liposome dilution buffer LDB1 (PBS [pH 7.2], 5% BSA, 1 mM 2-mercaptoethanol, 5 mM EDTA) at 23°C. Unbound liposomes washed out after 15 min to 1 h of incubation with 4 changes of LDB1. Images of rhodamine red fluorescent liposomes, EGFP-labeled A. fumigatus, and differential interference contrast (DIC)-illuminated cells were taken on microscope slides under oil immersion at a magnification of ×63 on a Leica DM6000B automated microscope. Five or six Z-stack images were recorded at 1-μm intervals and merged in Adobe Photoshop CC2018 using the Linear Dodge method. Bright-field and red and green fluorescent images were taken directly of cells on microtiter plates at magnifications of ×20 and ×40 on an Olympus IX70 inverted microscope and an Olympus PEN E-PL7 digital camera, and the bright-field and/or colored layers were merged in Photoshop.

### Cell growth and viability assays.

Liposomal stocks were stored at 900 μM AmB and diluted first 5- to 10-fold into liposome dilution buffer LDB2 (PBS [pH 7.2], 0.5% BSA, 1 mM 2-mercaptoethanol) and then into growth medium to achieve the desired final AmB concentrations. Control cells received an equivalent amount of LDB2. CellTiter-Blue cell viability assays were conducted as per the manufacturer’s instructions (Promega; document G8080) using 20 μl of resazurin reagent to treat 100 μl of fungal or animal cells in growth medium and incubating for 2 to 4 h at 37°C. Red fluorescence of electrochemically reduced resorufin product (excitation wavelength, 485 nm; emission wavelength, 590 nm) was measured in a Biotek Synergy HT microtiter plate reader. Data from six wells were averaged for each data point and standard errors calculated. Data for germination and hyphal length assays were collected manually from multiple photographic images taken at magnifcations of ×10 and/or ×20.
