# Erratum for Ambati et al., “Dectin-1-Targeted Antifungal Liposomes Exhibit Enhanced Efficacy”

**DOI:** 10.1128/mSphere.00121-19

**Published:** 2019-03-06

**Authors:** Suresh Ambati, Aileen R. Ferarro, S. Earl Kang, Jianfeng Lin, Xiaorong Lin, Michelle Momany, Zachary A. Lewis, Richard B. Meagher

**Affiliations:** a Department of Genetics, University of Georgia, Athens, Georgia, USA; b Department of Microbiology, University of Georgia, Athens, Georgia, USA; c Fungal Biology Group and Department of Plant Biology, University of Georgia, Athens, Georgia, USA

## ERRATUM

Volume 4, no. 1, e00025-19, 2019, https://doi.org/10.1128/mSphere.00025-19. Page 1: This article was published on 13 February 2019 with the third author's surname misspelled. The byline was updated in the current version, posted on 25 February 2019.

In the 2nd paragraph of the Materials and Methods section and in the legend to Supplemental Figure S1, the NCBI BankIT no. 2173810 should be replaced with GenBank accession no. MK322732.

