# Supplementary figures and images for: Dectin-1-Targeted Antifungal Liposomes Exhibit Enhanced Efficacy
Source: mSphere. 2019 Feb 13;4(1):e00025-19. doi: 10.1128/mSphere.00025-19 (PMC6374590; doi:10.1128/mSphere.00025-19)

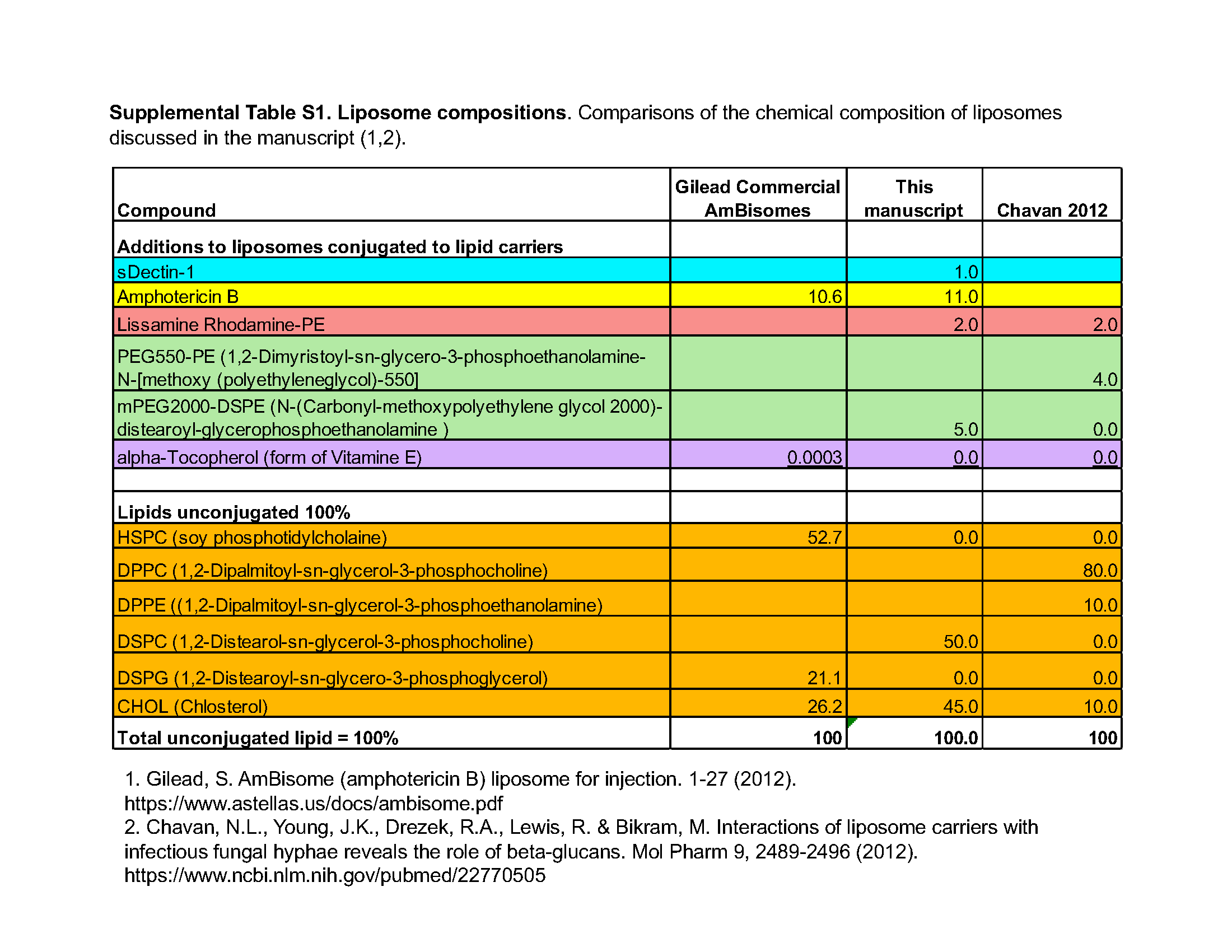

Supplement: TABLE S1 [file mSphere.00025-19-st001.tif]

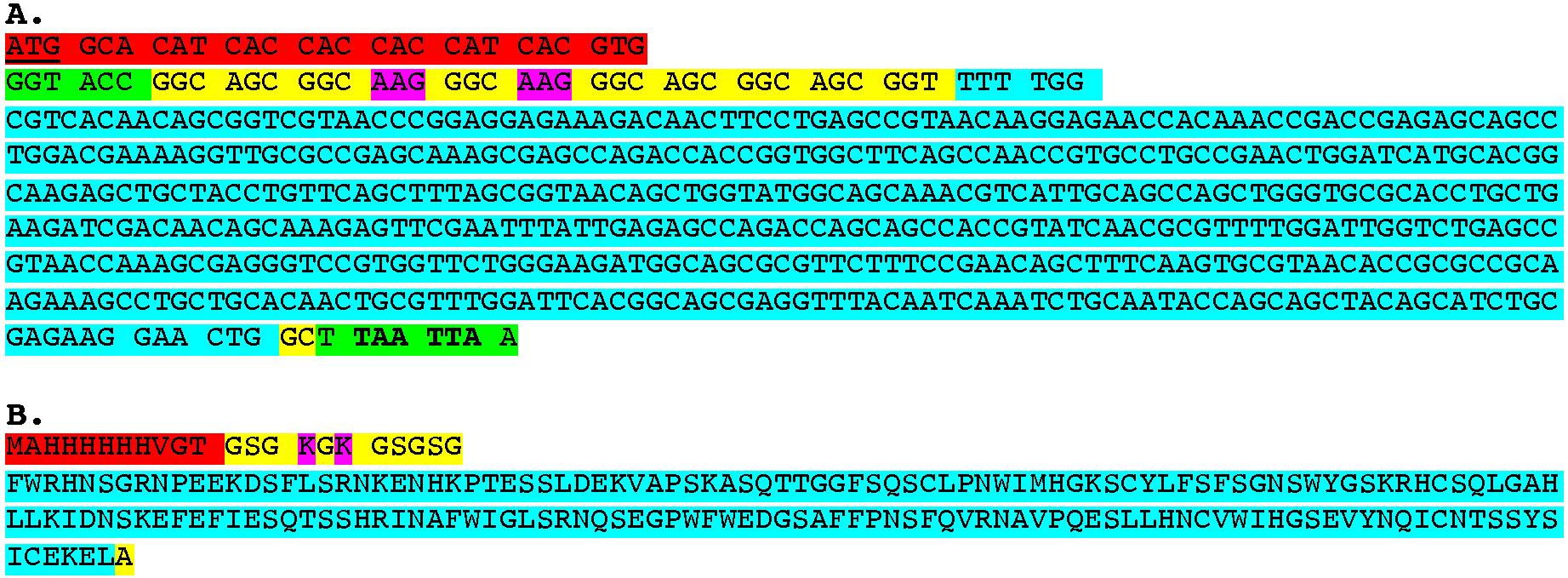

Supplement: FIG S1 [file mSphere.00025-19-sf001.tif]

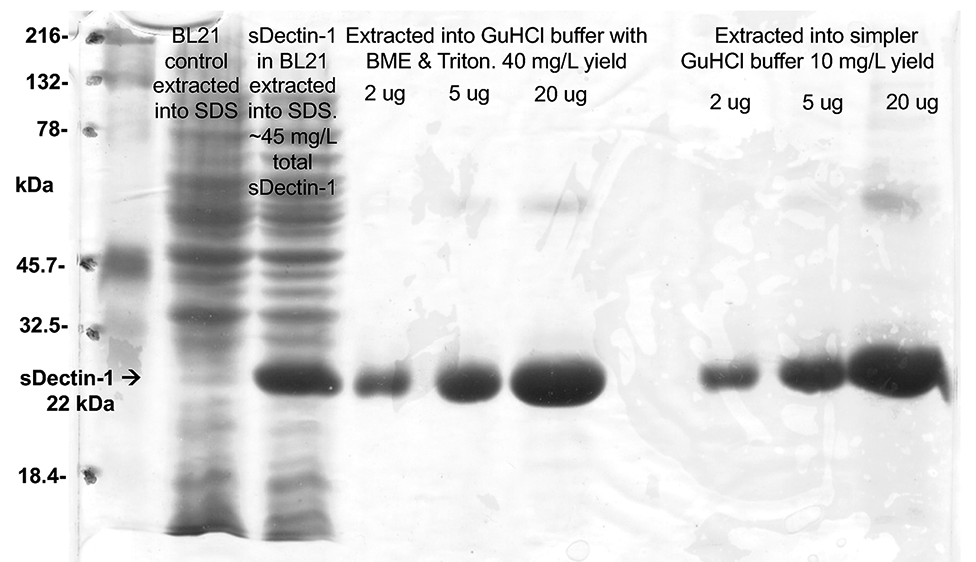

Supplement: FIG S2 [file mSphere.00025-19-sf002.tif]

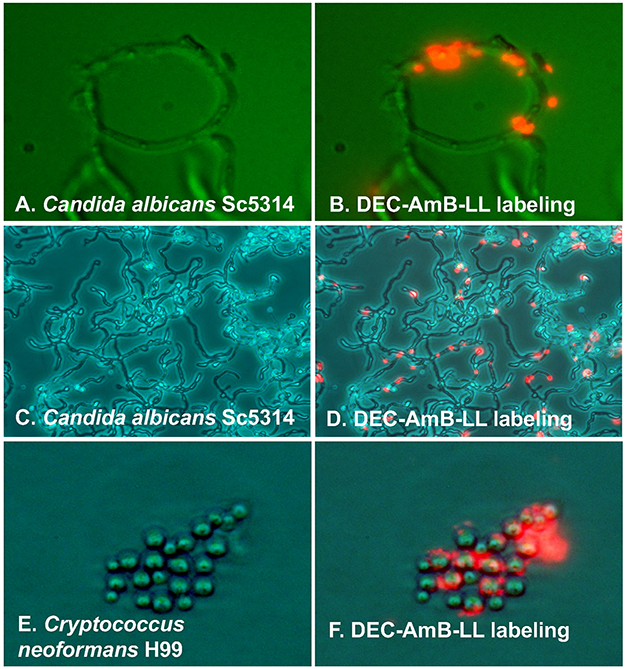

Supplement: FIG S3 [file mSphere.00025-19-sf003.tif]

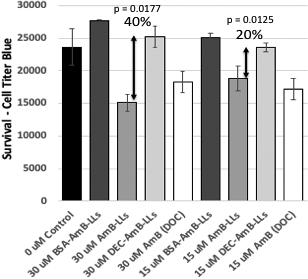

Supplement: FIG S4 [file mSphere.00025-19-sf004.tif]
